# Discovery of a Major QTL Controlling Trichome IV Density in Tomato Using K-Seq Genotyping

**DOI:** 10.3390/genes12020243

**Published:** 2021-02-08

**Authors:** Estefanía Mata-Nicolás, Javier Montero-Pau, Esther Gimeno-Paez, Ana García-Pérez, Peio Ziarsolo, José Blanca, Esther van der Knaap, María José Díez, Joaquín Cañizares

**Affiliations:** 1 Instituto Universitario de Conservación y Mejora de la Agrodiversidad Valenciana, COMAV, Universitat Politècnica de València, 46022 Valencia, Spain; esmani@posgrado.upv.es (E.M.-N.); esgipae@upv.es (E.G.-P.); ana.garcia@seeds4i.com (A.G.-P.); pziarsolo@uv.es (P.Z.); jblanca@upv.es (J.B.); mdiezni@btc.upv.es (M.J.D.); 2 Instituto Cavanilles de Biodiversidad y Biología Evolutiva, Universitat de València, 46980 Paterna, Spain; javier.montero@uv.es; 3 Institute of Plant Breeding, Genetics and Genomics, University of Georgia, Athens, GA 30602, USA; EsthervanderKnaap@uga.edu; 4 Department of Horticulture, University of Georgia, Athens, GA 30602, USA

**Keywords:** trichomes type IV, trichomes, tomato, QTL, K-seq, *Solanum pimpinellifolium*

## Abstract

Trichomes are a common morphological defense against pests, in particular, type IV glandular trichomes have been associated with resistance against different invertebrates. Cultivated tomatoes usually lack or have a very low density of type IV trichomes. Therefore, for sustainable management of this crop, breeding programs could incorporate some natural defense mechanisms, such as those afforded by trichomes, present in certain *Solanum* species. We have identified a *S. pimpinellifolium* accession with very high density of this type of trichomes. This accession was crossed with a *S. lycopersicum* var. *cerasiforme* and a *S. lycopersicum* var. *lycopersicum* accessions, and the two resulting F2 populations have been characterized and genotyped using a new genotyping methodology, K-seq. We have been able to build an ultra-dense genetic map with 147,326 SNP markers with an average distance between markers of 0.2 cm that has allowed us to perform a detailed mapping. We have used two different families and two different approaches, QTL mapping and QTL-seq, to identify several QTLs implicated in the control of trichome type IV developed in this accession on the chromosomes 5, 6, 9 and 11. The QTL located on chromosome 9 is a major QTL that has not been previously reported in *S. pimpinellifolium*. This QTL could be easily introgressed in cultivated tomato due to the close genetic relationship between both species.

## 1. Introduction

Agricultural production parameters like yield and quality are greatly affected by pests and diseases. Successful production is, therefore, highly dependent on pesticide application. Moreover, the use of pesticides imposes an additional production cost and can negatively impact human health and the environment. Additionally, pesticides can have negative effects on arthropod biodiversity, which in turn can result in loss of crop production [1]. As a consequence, new approaches are being sought in agricultural management programs to provide sustainable alternatives to pesticide applications [2]. Recent efforts to enhance sustainability have focused on the development of crops with genetic resistance to various pests and diseases.

The presence of trichomes is an important morphological defense against insect pests [3,4]. Specifically, glandular trichomes that accumulate and exude secondary metabolites, are known to interfere with oviposition, larval fixation and development, while also trapping or poisoning insects [2,5,6]. The type of trichomes and stored compound depends on the species and could include acylsugars, methyl ketones, terpenoids, phenylpropenes or flavonoids [7]. Breeding for resistant cultivars with an increased density of glandular trichomes is a valuable option to reduce the impact of arthropod pests. However, this strategy requires a detailed knowledge of the genetic control and molecular basis of trichome development.

Trichomes are a common morphological defense in the genus *Solanum* [8,9]. Eight types of trichomes have been described on the stems of plants of this genus [10] type I, IV, VI and VII are glandular trichomes. These glandular trichomes accumulate different compounds—Type I and IV, acylsugars; type VI, monoterpenes and sesquiterpenes and type VII, alkaloids—and provide different levels of protection against pests [2]. In particular, the presence and density of type IV glandular trichomes—trichomes with a short multicellular stalk and small gland at the tip [11]—has been associated with pest resistance such as spider mite [12,13], whitefly [14,15,16,17] and potato moth [18]. Moreover, resistance against whiteflies and aphids has been associated with the production of acylsugars in this type of trichome [19]. Cultivated tomato (*Solanum lycopersicum* var. *lycopersicum* L.) typically lacks or has a very low density of type IV trichomes [2]. Thus, specific breeding programs that will incorporate these natural defenses that are common within the *Solanum* genus, may improve crop management against a host of arthropod pests.

Several wild relatives of tomato have shown to be a rich source of genetic diversity for type IV trichomes. Particularly *Solanum pennellii* Correll, *Solanum habrochaites* S. Knapp & D.M Spooner and *Solanum galapagense* S.C.Darwin & M.I. Peralta display trichome densities higher than 90 trichomes/mm^2^ [17,20,21]. Inheritance of type IV trichome density has been studied using interspecific crosses among these related species and tomato. The presence of trichome type IV on *S. pennellii* has been proposed to be under the control of two dominant unlinked genes [22]. Andrade et al. [17] found that inheritance of trichome type IV on *S. galapagense* is relatively simple and could be controlled by incomplete recessive alleles. Alternatively, genetic factors underlying type IV trichome density have been identified by quantitative trait locus (QTL) mapping studies in other studies. Some QTLs have been confirmed on chromosome 2 of *S. pennellii* [21], in chromosome 2 and 9 of *S. habrochaites* [23] and *S. galapagense* [24]. Synthesis of acylsugars has also been associated with QTLs in populations derived from *S. pennelli.* These QTLs were located on chromosomes 2, 3, 4, 5, 6 and 11 of *S. pennellii* [21,25,26].

Despite the fact that these wild species are described as a potential source of type IV trichomes density, their use in breeding programs is limited because the sexual incompatibility hinders their introgression and due to linkage drag resulting in undesirable agronomic traits such lower fruit size or higher morphological heterogeneity in addition to increased type IV trichome density. As a consequence, a more closely related wild species like *Solanum pimpinellifolium* L., a red-fruited, facultative self-compatible species is generally preferred in tomato breeding programs as excessive linkage drag can be avoided in backcrossing programmes. Unfortunately, the number of accessions of *S. pimpinellifolium* with a relatively high density of type IV trichome reported to date is scarce. Rahka et al. [16] described accessions that were characterized by densities lower than 4.5 trichomes/mm^2^. Fernández-Muñoz et al. [27] found a *S. pimpinellifolium* accession (TO-937) characterized by a mean of 16.3 type IV trichomes/mm^2^ in the abaxial surface, and Firdaus et al. [28] found a *S. pimpinellifolium* accession (LA1584) with 21.0 trichomes/mm^2^. Interestingly, a population derived from the accession TO-937 confirmed two QTLs in chromosome 2 conferring resistance to spider mites [29].

Trichome presence and density is also known to depend on developmental factors such as plant and leaf age. Vendemiatti et al. [30] found a decrease in type IV trichomes density from cotyledons (40 trichomes/mm^2^) up to sixth leaves (<5 richomes/mm^2^) in commercial cultivars of tomato. *S. habrochaites* showed higher pubescence in 9-week old plants compared to 6-week old plants (18 vs. 6 type IV trichomes/mm^2^) [31]. F2 and F1 populations between *Solanum lycopersicum* and *S*. *pennellii* also exhibited higher densities in plants at 10 and 13 weeks after transplanting, compared to 7 weeks aged plants [32]. However, Wilkens et al. [33] described a reduction of density of type IV trichomes with plant age in an older Ecuadorian tomato cultivar. An effect of light was described in *S. habrochaites,* trichome densities were higher under a photoperiod of 8 h of light, compared to 14–15 h [34].

The main objective of this study was to map the quantitative trait loci controlling type IV trichome density in a *S. pimpinellifolium* accession (BGV016047) that appears to present the highest density of type IV trichome (>100 trichomes/mm^2^) to be described for this species. Populations derived from this accession using two different genetic backgrounds have been used to map the loci controlling the presence and density of type IV trichomes on chromosomes 9 and 11. We demonstrate the practical use of K-seq, a new genotyping technology based on the amplification with the Klenow polymerase of genomic regions with short oligonucleotides, followed by standard PCR and Illumina sequencing [35].

## 2. Materials and Methods

### 2.1. Plant Material

During the characterization of the Varitome collection (https://solgenomics.net/projects/varitome) of *S. pimpinellifolium* (27 accessions), *S. lycopersicum* var. *cerasiforme* (121) and *S. lycopersicum* var *lycopersicum* (15) from Perú, Ecuador and México [36], an accession of *S. pimpinellifolium* (BGV016047) with a high density of glandular type IV trichomes was found.

To study the genetic basis of trichome IV density, we have used the F1 and F2 collections developed by the Varitome project [36]. BGV016047 (also coded as PAS014479 in Varitome) was crossed with two different genetic backgrounds with low trichome density: *Solanum lycopersicum* var. *cerasiforme* LA2278 (LA2278 × BGV016047, referred hereafter as SLC family) and *Solanum lycopersicum* var. *lycopersicum* cv. Moneymaker (MoneyMaker × BGV016047, referred hereafter as SLL family). F1 populations were developed using BGV016047 as the male parent in both families, and the F2 generation was obtained by self-pollination. BGV016047 was chosen as the male parent because flowers of *S. pimpinellifolium* are smaller and more difficult to emasculate. Additionally, for this study the F1 of SLL family was also backcrossed with Moneymaker (BC1).

All accessions used in this paper were provided and are available at the genebank of Instituto de Conservación y Mejora de la Agrodiversidad Valenciana (COMAV) at the Polytechnic University of Valencia and at the Tomato Genetics Resource Center (TGRC, Davis, CA, USA).

### 2.2. Glandular Type IV Trichome Density Characterization

To evaluate the effect of plant age on glandular type IV trichome density in the BGV016047 accession, an experiment was conducted during spring/summer 2018. The effect of plant age was evaluated by measuring trichome density on leaflets on the same developmental stage. Ten plants were grown in the greenhouse and fertigated with a nutrient solution containing 14 mM NO_3_^−^, 1 mM H_2_PO_4_^−^, 2 mM SO_4_^2−^, 1 mM NH_4_^+^, 16 mM K^+^, 4 mM Ca^2+^, 2 mM Mg^2+^, 15 µM Fe^2+^, 10 µM Mn^2+^, 5 µM Zn^2+^, 30 µM B^3+^, 0.75 µM Cu^2+^ and 0.6 Mo^6+^ [37]. After transplanting, the second leaflet of the second fully expanded leaf from the apex of the plant was sampled weekly from day 9 to 89 after transplanting (13 sample points). Leaflets were removed from the plant and placed in a Petri dish with wet filter paper to prevent desiccation. Leaflets were cut, perpendicularly to the central vein, into two approximately equal sections to facilitate the observations. These sections were viewed under a 40× magnification microscope (Alphasot YS-2, Nikon, Tokio, Japan) and trichome density was estimated in four random areas of 1 mm^2^ per leaflet. Counts of type IV trichomes were made on the abaxial surface of each leaflet, avoiding the interveinal areas and the main vein where trichome density was higher (Supplementary Figure S1).

The influence of leaf age was explored by following the same leaves over time. Three leaves on 10 different plants were followed during three consecutive weeks, starting when the leaves were in the second position from the apex (at 49, 53 and 60 days after transplanting, respectively). Type IV thricome density was measured as described above.

Trichome density was evaluated in 195 F2, 50 BC1 and controls (24 BGV016047, 10 MM, 16 F1) for the SLL family and in 151 F2 and controls (10 BGV016047 and 10 LA2278) for the SLC family. Seeds were sown in trays with commercial potting mix and transplanted to the greenhouse in a completely randomized design five weeks (SLC family) or four weeks (SLL family) after sowing in Paiporta (Valencia, Spain). The phenotyping started once the leaves from BGV016047 exhibited a density higher than 10 trichomes/mm^2^. As a consequence, the SLC family was evaluated the sixth, seventh and eighth weeks after transplanting, whereas the SLL family was evaluated the third, fourth and sixth weeks after transplanting. Type IV trichome density was measured on leaflets from leaves in the second position below apex, following the procedure described above. To reduce the environmental, plant and leaf age effects, measurements of the whole family were made during the same week. SLC family was phenotyped during spring/summer 2018 and SLL family during autumn/winter 2017.

### 2.3. DNA Extraction and Plant Genotyping by K-Seq

For the SLC family, a total of 137 F2 individuals were genotyped out of the 151 that were phenotyped. Additionally, 15 parental controls of this family were also genotyped. In the case of the SLL family, a Bulk Segregant Approach was used: F2 plants with the highest and lowest trichome density were chosen and individually genotyped 15 plants with an average density higher than 10 trichomes/mm^2^ and 15 plants with a density lower than 0.5 trichomes/mm^2^. Genomic DNA was extracted from leaves using a CTAB protocol [38].

Genotyping was performed using K-seq, a novel genotyping method based on the reduction of genome complexity using Klenow polymerase and short oligonucleotides [35]. The genotyping was done as described in Ziarsolo et al. [35] but using 25·°C as annealing temperature for the Klenow polymerase amplification. A total of 200 ng of DNA of each individual was subjected to two cycles of amplification using Klenow polymerase and short primers. After each cycle, an Exonuclease I digestion step was performed to remove uncopied genomic DNA and free primers. Finally, the double strand fragments were amplified during 13 cycles using a standard PCR reaction with indexed primers. Short oligonucleotides and PCR primers are shown in Supplementary File 1. PCR products of each individual were pooled in two pools using the same volume PCR sample, size selected and sequenced in two Hiseq2500 lanes 2 × 150 pb (Illumina, San Diego, CA, USA) by the CNAG-CRG (Barcelona, Spain). The reads have been deposited under Bioproject PRJNA649673.

Raw reads quality was inspected using FastQC v. 0.11.5 [39] and low quality reads and adapters were removed using Trimmomatic v. 0.36 [40]. Clean reads were mapped against *Solanum lycopersicum* genome build SL2.5 [41] using BWA-MEM v. 0.7.17 [42]. SNP calling was performed using freebayes v 1.3.1 [43] and the SNPs with more than 50% of missing data and a minor allele frequency lower than 0.05 were filtered out those using custom Python scripts that are publicly available at https://github.com/bioinfcomav.

### 2.4. Genetic Mapping, QTL and QTL-Seq Analysis

QTL analysis was performed using F2 individuals of SLC family using two different approaches: (1) construction of a genetic linkage map followed by QTL mapping, and (2) QTL-seq analysis [44] between F2 individuals with extreme trichome density values for each mapping population.

For the first approach, the filtered SNPs were transformed into ABH genotype coding using custom scripts for the 107 F2 individuals of the SLC family that passed the quality filters. Transformed genotype data was corrected using GenotypeCorrector [45] using a sliding window size of 41 SNPs and after binning consecutive homozygous markers. Construction of the genetic map and QTL location was performed with *R/qtl* [46] and *ASMap* [47] packages from R software. For the construction of the genetic map, adjacent SNPs with the same genotype were also binned and SNPs in chromosome 0 were discarded. QTL mapping was done by single interval mapping based on a non-parametric test (Kruskal-Wallis test). Statistical significance thresholds were calculated based on 1000 bootstrap. QTL location interval was established based on 1.5 LOD units drop. Additionally, the effect of each QTL was determined by fitting to a generalized linear model (logistic model) assuming a negative binomial distribution and a log link function using R package *MASS* [48]. Best fitting distribution for trichome density was found using the R package *fitdistrplus* [49]. Goodness of fit was checked for each model. Proportion of deviance (*D*^2^) explained by the model and Nagelkerke *R*^2^ were calculated using functions “Dsquared” and “RsqGLM” from the *modEvA* package [50].

The QTL-seq was done by bulking the genotype information of F2 individuals with the lowest and the highest type IV trichome average density. For the SLC family, 15 individuals with an average density of 0.05 trichomes/mm^2^ (range from 0 to 0.25 trichomes/mm^2^) and 15 with an average density of 94.52 trichomes/mm^2^ (range from 53.00 to 212.50 trichomes/mm^2^) were selected. For the SLL family, 15 individuals with an average density of 0.03 trichomes/mm^2^ (range from 0 to 0.25 trichomes/mm^2^) and 13 with a density of 28.35 trichomes/mm^2^ (range from 11.25 to 64.25 trichomes/mm^2^). Allele counts for each group were summed, SNPs with a read depth higher than 150 for SLC family and 250 for SLL family were filtered out. Additionally, SNPs with a reference allele frequency lower than 20% or higher than 80% were also removed. For each bulk, a SNP-index per loci was calculated as the percentage of reads containing a different allele from the reference genome. The delta (Δ) SNP-index was calculated by subtracting the SNP-indices of the bulks at each loci [44], and candidate QTL regions were identified by using a 1 Mb sliding window. Confidence intervals for the ΔSNP were determined using 1000 simulations. QTL-seq analyses were performed with the QTLseqr R package [51].

Additionally, we also performed an association analysis along the genome for type IV trichome density. We explored the association between genotype for each marker and trichome density by fitting a logistic regression assuming a negative binomial distribution as above. Resulting *p*-values for each marker were adjusted for multiple comparison by using Benjamini-Yekutieli procedure [52] in R software v.3.6 [53].

## 3. Results

### 3.1. Type IV Trichome Density Characterization of S. pimpinellifolium Accession BGV016047 with a High Density of Type IV Trichomes

**Figure 1:**
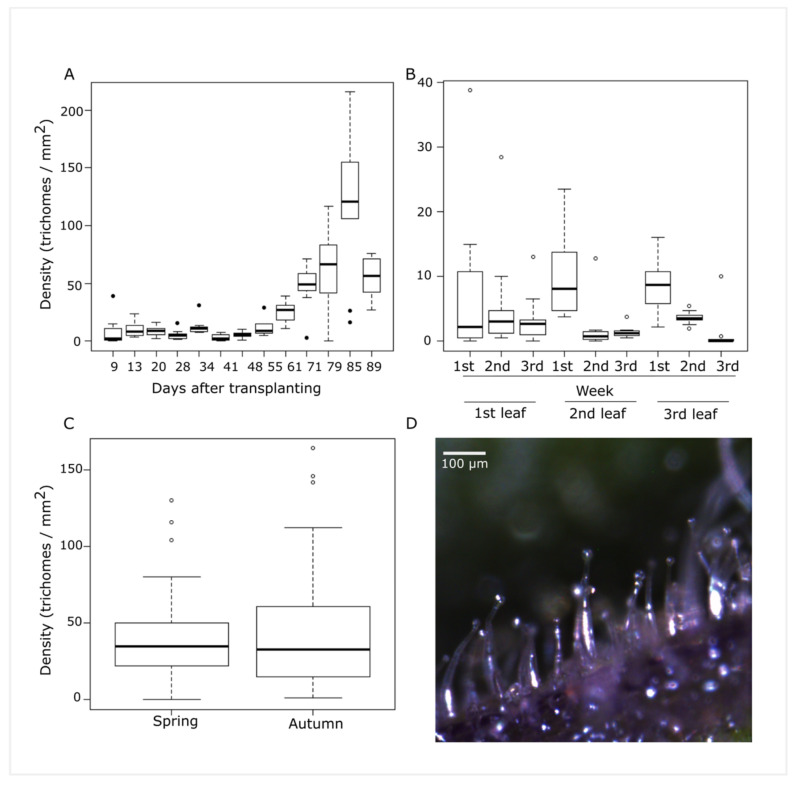
Effect of plant age (**A**), leaf age (**B**) and growing season (**C**) on type IV trichome density. Photography of trichomes type IV (**D**).

Density of type IV trichomes varied with the age of the plant (Figure 1A,D). The density of trichomes in the second leaf from the apex ranged from a median of 0 to 9.5 trichomes/mm^2^ (range from 0 to 43 trichomes/mm^2^) until day 48 after transplanting, afterwards trichome density started to increase to a maximum median of 118 trichomes/mm^2^ (range 8 to 240 trichomes/mm^2^). Trichome density also showed an increased variability as the plant grew older. In the last week of the experiment (day 89 after transplanting), a drop in the trichome density was observed (median of 57.5 trichomes/mm^2^). This drop coincided with an increase of minimal daily temperature and with the death of two analyzed plants, so it could be due to environmental factors. The effect of leaf age on density was measured by following three leaves during three consecutive weeks which corresponded with their location as second, third and fourth leaf from the apex (days 49, 53 and 60 after transplanting). This result (Figure 1B) showed that trichome density on leaflets from young leaves decreased as the leaflet was expanding in the 2nd and 3rd leafs. Trichome density was evaluated in two different seasons (spring/autumn) (Figure 1C). Trichome density during the first week of phenotyping, when parent plants have reached a density of 10 trichomes/mm^2^ in at least one measure, was similar between seasons (median of 35 vs. 34 trichomes/mm^2^), although with a higher variability in autumn (interquartile range 27 in spring vs. 46 in autumn).

### 3.2. Trichome Type IV Density on Segregating Populations

**Figure 2:**
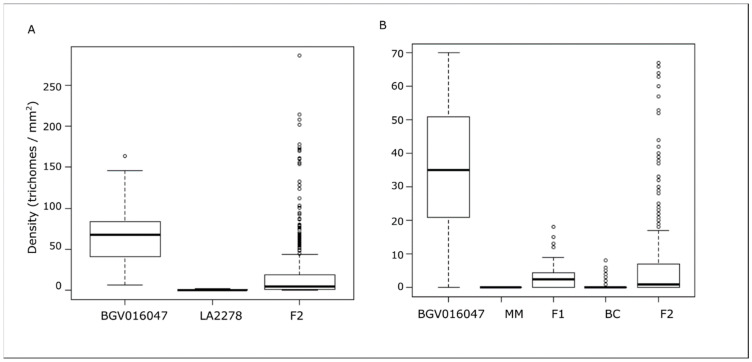
Density of type IV trichomes in the segregating populations for the last week of phenotyping for the SLC family (BGV016047 × LA2278) (**A**) and SLL family (BGV016047 × MoneyMaker) (**B**).

The *S. pimpinellifolium* accession BGV016047 was crossed with low trichome density *S. lycopersicum* var. *cerasiforme* accession LA2278 and *S. lycopersicum* var. *lycopersicum* ‘MoneyMaker’ (Supplementary File 2). Figure 2 shows the density distributions of the different populations derived from these crosses. The low trichome density parents exhibit either none or a very low trichome density, whereas the F2 for both families showed values that were skewed towards the low-density parent. However, in some cases some F2 reached or even exceeded the densities of BGV016047. F2 individuals of the SLL family (Figure 2B) showed a higher average density than the corresponding F1 (5.5 vs. 3.6) but a lower median (1 vs. 2.5), indicating a distribution shifted towards lower or higher values than the F1. The backcross generation (BC) obtained from the backcross F1 × MoneyMaker exhibited a very low number of trichomes, recovering the MoneyMaker phenotype. Altogether, this pattern of segregation suggests that the character could be under control of a few QTLs. This pattern did not change with plant age as the same relationship was observed on each of the three weeks that were phenotyped (Supplementary Figure S2).

### 3.3. QTL and QTLseq Analysis in BGV016047 × LA2278 Population

The number of raw reads per sample obtained in the K-seq genotyping varied from 399,182 to 7,287,426. This variability was likely due to the fact that the pool of samples was made using equal volumes of PCR product of each sample and not quantifying them. In Supplementary Figures S3–S5 a summary of K-seq read number, mapping and SNP calling results is shown. The average number of raw reads was 2.4 million reads per sample, 1.8 million reads per sample could be mapped against the reference genome [41] with a mapping quality higher than 57 (75.68% of raw reads), which resulted in an average genome coverage of 6.97% at read depth 1 or higher (1.28% at depth 5 or higher and 0.60% at depth 10 or higher). After including the parental genome sequences as references into the SNP calling analysis, a total of 4,993,950 SNPs were obtained. The number of SNPs found in the K-seq genotyped samples varied from 121,019 to 729,686. After removing SNPs with a minor allele frequency lower than 5%, 4,136,452 SNPs were kept and 308,400 were finally retained after removing those with 50% of missing data. Out of those, 147,326 SNPs could be transformed to ABH coding. 107 F2 samples were finally used to build the genetic map for the F2 population (Supplementary File 3). Adjacent SNPs with the same parental genotype were binned in 8285 bin markers. Markers were grouped in 12 linkage groups (Table 1 and Supplementary Figure S6) that resulted in a total map length of 1600.4 cm with an average of 12,277.2 SNPs and 690.4 bin markers per chromosome, and an average length of 133.4 cm per chromosome with an average distance of 0.2 cm.

**Figure 3:**
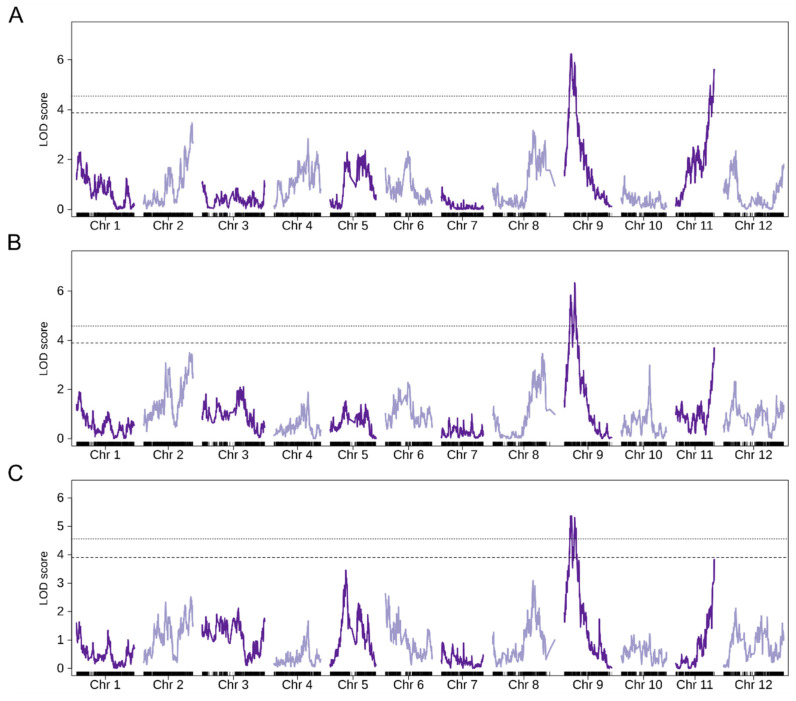
Results of non-parametric single interval mapping QTL analysis for each of the three weeks phenotyped. (**A**) 6th week after transplanting, (**B**) 7th week and (**C**) 8th week.

**Figure 4:**
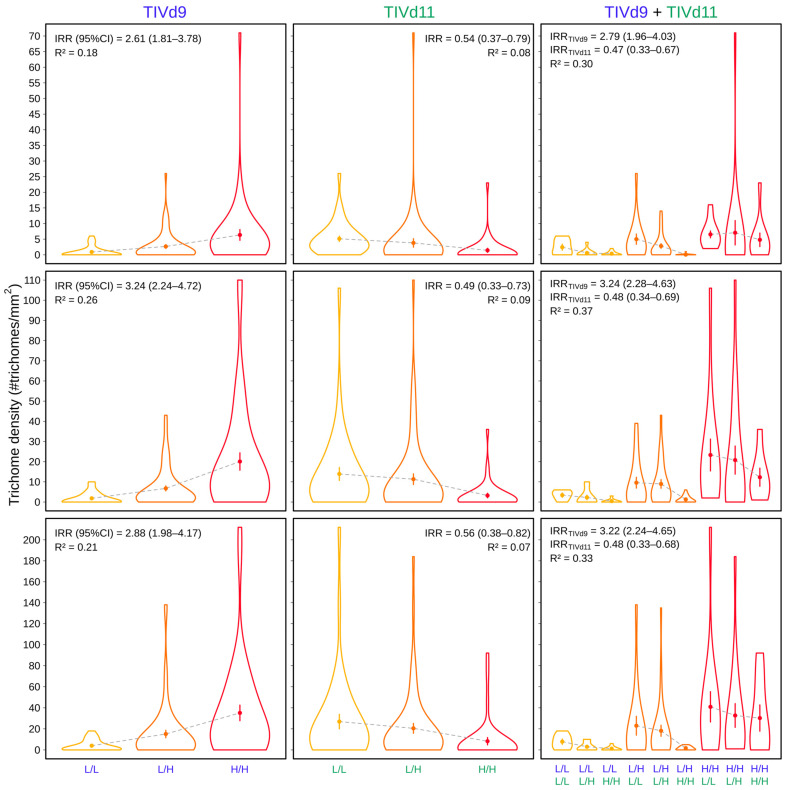
Effect of QTLs TIVd9, TIVd11 and the additive model on trichome density along the three phenotyped weeks (6th, 7th, 8th weeks after transplanting. Incidence rate ratio (IRR) and 95% confidence intervals (values between brackets) and Nagelkerke R^2^ of the negative binomial logistic regression are shown for each combination. Violin plots for the two homozygotes (L/L and H/H) and the heterozygote (L/H) are represented. L refers to an allele of the low trichome density parental (LA2278) and H to an allele of the high trichome density parental (BGV016047). Mean trichome density and standard error for each genotype is shown inside each violin plot.

The non-parametric QTL mapping of BGV016047 × LA2278 population showed two significant QTLs in chromosomes 9 and 11 (Figure 3 and Table 2) named as TIVd9 and TIVd11. As trichome density varied with plant age, the variance and significance of both QTLs also varied through time, however TIVd9 was significant in all three sets of phenotyping data. Explained variance by the negative binomial logistic regression model exclusively by TIVd9 ranged from 0.18 to 0.26 based on Nalgerke’s *R*^2^, and from 0.30 to 0.37 if both QTLs were considered in an additive model (Figure 4 and Table 3). The incidence rate ratio (IRR) of the logistic model shows that in the TIVd9 locus each additional allele from the high-density parent increased the expected trichome IV density by 3 (IRR varied from 2.61 to 3.24 depending on the analyzed week), so a homozygote bearing the two SP alleles has a 9 fold expected density. Whereas in TIVd11, each allele of the BGV016047 parent resulted in a decrease of the expected trichome density of about 50% (IRR varied from 0.48 to 0.54).

As the QTL mapping was performed using a single interval mapping based on a non-parametric method, we decided also to test the association by adjusting to a negative binomial distribution, thus we fitted the density of type IV trichomes with a logistic regression using genotype data as a explanatory factor (Table 2 and Supplementary Figure S7). After adjusting for multiple comparisons, the QTL in chromosome 9 appeared in all three weeks of phenotyping, coinciding with the TIVd9 region. Interestingly, no region in the chromosome 11 reached a significant association in any case (Supplementary Figure S7). However, an additional QTL was found when using the phenotypic data of the third week at the beginning of chromosome 6 (TIVd6). This QTL was able to explain a 22% of variance according to Nalgerke’s *R*^2^ when considered in a model with a single factor or 32% when considered in an additive model together with TIVd9 (Table 3). IRR for TIVd6 and TIVd9 was 1.86 and 2.16 respectively in the additive model.

**Figure 5:**
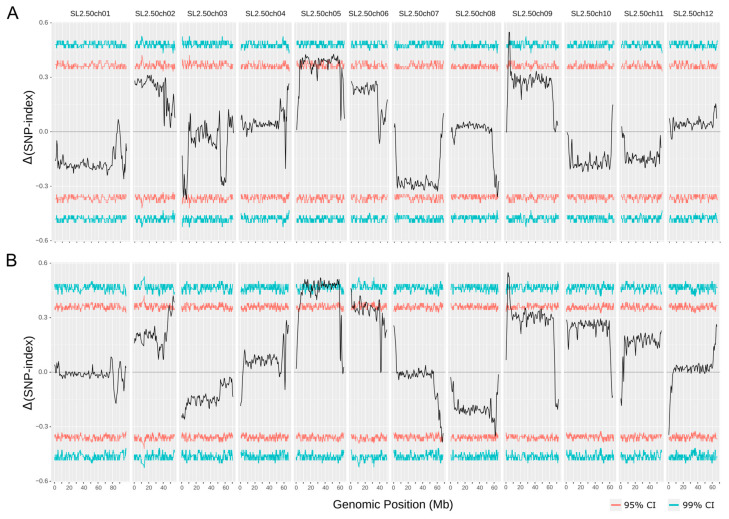
Distribution of Δ(SNP-index) along the chromosomes based on 1 Mb windows for the (**A**) SLC family (BGV016047 × LA2278) and (**B**) SLL family (BGV016047 × MoneyMaker). The 95% and 99% confidence thresholds are shown.

A QTLseq analysis comparing F2 individuals with high and low trichome density was also performed. A total of 124,514 SNPs were retained after filtering by depth and MAF. Results show a significant QTL at 99% CI in chromosome 9 overlapping with the location of TIVd9 (Table 2 and Figure 5A). Additionally, most part of chromosome 5 seems to present a significant deviation of the ΔSNP index above the 95% confidence interval.

### 3.4. QTL Validation Using S. lycopersicum var. lycopersicum as Genetic Background

The accession BGV016047 was also crossed with MoneyMaker, a *S. lycopersicum* var. *lycorpersicum* accession, in order to test if the same genetic control was maintained with a different genetic background. A QTLseq analysis was performed on the F2 of SLL family by bulking individuals with the highest and lowest trichome IV density. A total of 107,112 SNPs were used after filtering by genotype depth and MAF. The results show that the QTL in chromosome 9 was also detected and it overlaps the region detected in SLC family (Table 2 and Figure 5). Chromosome 5 also presented the same pattern that in the cross with *S. lycorpersicum* var. *cerasiforme,* although the signal was stronger. Additionally, a region at the beginning of chromosome 6 also crossed the 99% CI threshold. This region overlaps with the signal found for SLC family in the association analysis when using phenotype data of the 8th week after transplanting. Other regions in chromosome 2, 7 and 8 showed some signal above the 95% confidence interval.

### 3.5. Candidate Genes

The region of TIVd9 (SL2.50ch09: 1,966,640–4,371,756 bp) comprises 248 genes (Supplementary Table S1), which include two MYB transcription factors Solyc09g010820, a MYB R3 transcription, and Solyc09g009450, a Myb/SANT-like protein paralogous of *Arabidopsis* AT2G24960. The MYB family is involved in development and cell division. Additionally, two trichome birefringence-like proteins are located (Solyc09g010260 and Solyc09g010270) close to the location where the maximum significance is found (about 3.7 Mb). This gene family has been shown to regulate the density of trichomes [54,55].

Besides these genes related to regulation of expression and cell division, other genes in this region of chromosome 9 are linked to genes related to the production of secondary metabolites that accumulate in glandular trichomes [56]: genes related to acylsugar production like acyltransferases (Solyc09g008520) and glycosyltransferases (Solyc09g008510, Solyc09g009010, Solyc09g010760), fatty acid synthesis like fatty acyl-CoA reductase (Solyc09g009570, Solyc09g009580) and phosphopantetheinyl transferase family protein (Solyc09g009960) or transporters like ABC transporters (Solyc09g009910) that are involved in lipid and secondary metabolites transport. Additionally, other genes related to plant-defenses mechanisms that also are known to be upregulated in glandular trichomes like subtilisin-like protease (Solyc09g009750) are also found.

In the region of TIVd11 (SL2.50ch11:54,298,873–56,297,460 bp), two out of 265 genes are transcription factors of the family MYB R2R3, Solyc11g072060 paralogous of *Arabidopsis* MYB104 and Solyc11g073120 paralogous of MYB58, and an Agamous-like MADS-box protein (Solyc11g069770). Additionally, the gene for gibberellin 20-oxidase-3 (Solyc11g072310) is also located in this region. This enzyme plays a central role on the synthesis of gibberellins which have a positive effect on trichome formation [57]. Other genes related to acylsugars metabolism that are found in TIVd11 region are Solyc11g072980 and Solyc11g072990 that code for a 3-ketoacyl-CoA synthase, a long fatty acid dehydratase (Solyc11g073130), ABC transporters (Solyc11g069710 and Solyc11g069820), a glycosyltransferase (Solyc11g071230) and an acyltransferase (Solyc11g069680). Interestingly, this last gene presents an SNPs that was classified as a high impact SNPs by SNPEff.

In the detected region of chromosome 6 (from 8008 to 943,353 bp and 95 genes) three more MYB transcription factors are found, two paralogs of *Arabipodis* MYB48 (Solyc06g005310 and Solyc06g005320) which is related to the synthesis of flavonoids that also accumulate in type IV trichomes and a paralog of MYB59 (Solyc06g005330) which is involved in the regulation of cell cycle progression and root growth. The region of chromosome 5 covers a high proportion of this chromosome and includes 843 genes (from 9,189,958 bp to 59,293,229) (Supplementary Table S1). Several genes related to acylsugars are located in this region like several acyltransferases (Solyc05g025890, Solyc05g039950, Solyc05g047610, Solyc05g047640, Solyc05g016030), long chain acyl-CoA synthetase 2 (Solyc05g041520) or ABC transporters (Solyc05g018510, Solyc05g023940). Also defense related proteins like glutathione S-transferase (Solyc05g026210), kirola-like (Solyc05g046140, Solyc05g046150, Solyc05g046160, Solyc05g046170, Solyc05g046210, Solyc05g046220) or FLOWERING LOCUS D (Solyc05g016300) that are upregulated in trichomes [56]. Besides that, transcriptions factors including a Myb/SANT-like (Solyc05g018830) and two MADS-box transcription factor (Solyc05g015720 and Solyc05g015730) are also located in this region as well as a protein trichome birefringence-like (Solyc05g019980).

## 4. Discussion

The presence of glandular trichomes, especially type IV trichomes, is involved in arthropod pest resistance, which is a desirable trait for sustainable agriculture. Glandular type IV trichomes have been described in wild *Solanum* species such as *S. habrochaites, S. pennellii, S. galapagense, S. cheesmaniae* and *S. neorickii,* with accessions reaching densities higher than 70 trichomes/mm^2^ [17,20,21,24]. Unfortunately, these species are distant relatives from cultivated tomatoes and their use as sources for tomato improvement is limited due to the incorporation of undesirable characters. *S. pimpinellifolium* is the closest wild relative of cultivated tomato and a common source of alleles for breeders. However, until now, presence of type IV trichomes has only been described at low densities (<20 trichomes/mm^2^) [28,58,59]. In the present study, we have characterized a *S. pimpinellifolium* accession (BGV016047) with a high density of type IV trichomes (from 9.5 trichomes/mm^2^ at 48 days after transplanting, to a maximum median of 118 trichomes/mm^2^) that could be of great interest for tomato genetic improvement. Density of this type of trichomes is known to be dependent on developmental conditions, such as leaf and plant age [31]. Accordingly, we have observed a density increase with plant age, reaching maximum trichome densities of 240 trichomes/mm^2^ in young leaves (118 trichomes/mm^2^ maximum median density) 12 weeks after transplanting, while trichome density tends to decrease as leaves continue growing and the foliar area increases. Besides plant and leaf age, some degree of variability has been observed between measures and seasons, which is likely to be the result of environmental conditions or resources availability [31,33].

K-seq is a reduced representation library sequencing method that provides a high-throughput and cost-effective genotyping alternative to existing methods. K-seq genotyping achieves enough genome coverture and depth for many genotyping needs and produces a high number of SNPs. We have used the K-seq genotyping to develop an ultra-dense genetic map with almost 150 thousand SNPs. K-seq could be easily adapted to different necessities varying the primer number and the K-mer sequences [35]. In this study, we have genotyped 96 samples per Hiseq2500 lane, resulting in a low cost by sample. However, sample cost could be further reduced using more multiplexing indexes and reads by lane as the number of SNPs obtained exceeds the needs for the development of a detailed F2 map. So, K-seq is a cheap and easy methodology to genotype that can be used by any laboratory.

Using K-seq we have built an ultra-dense genetic map with 147,326 markers at an average distance between markers of 0.2 cm that allowed us to perform a detailed mapping of the phenotype density of type IV trichomes in two F2 populations and to identify genes candidates for this trait. The inheritance of type IV trichomes has been previously addressed in other accessions and species, but the genes are still unknown. More information is available regarding QTLs related to insect resistance and metabolism of acylsugars, the most common chemical accumulated in type IV glandular trichomes, that also affects density and development of trichomes [21,25,26,60,61,62]. In *S. galapagense* and *S. habrochaites* the density of type IV trichomes was considered to be controlled by an incompletely recessive allele in a major locus [17,63]. In *S. pennellii* and *S. pimpinellifolium,* results supported two dominant unlinked genes [22,27,32]. The pattern of inheritance found in the two segregating families derived for this study would also support the involvement of a limited number of genes making feasible their management in a breeding program.

By using the information of the two populations derived from crosses between the accession BGV016047 and two different genetic backgrounds, we have detected a new major QTL at the beginning of chromosome 9 (TIVd9) in *S. pimpinellifolium,* which accounts up to 26% of the explained variance. Each allele increased the expected trichome IV density by 3. Additionally, a minor QTL has been found in chromosome 11 (TIVd11) and likely in chromosomes 6 and 5. The major QTL TIVd9 has not been previously detected in *S. pimpinellifolium,* where only two QTLs likely involved in trichome formation have been described in the telomeric region of chromosome 2 [29]. This region in chromosome 9 has also been linked to type IV trichome density in *S. galapagense* although the percentage of explained variance was much lower (2.8–8.3%) [64]. Another region in chromosome 9, adjacent to TIVd9 and with minor effects on trichome density, has also been detected in *S. galapagense* [24]. In *S. habrochaites*, QTLs for type IV trichome density in chromosome 9 have also been described. In this species, three QTLs for trichome density in chromosome 9, 10 and 11 were detected, all together explaining the 22% of the variance [23]. The QTL on chromosome 9 overlaps with our QTL TIVd9, although explains a lower percentage of the variance (11%) than in our study. Interestingly, this QTL is also associated with a reduced oviposition by *Bemisia tabaci.* The QTL on chromosome 11 does not overlap with our QTL as it was located 4Mb away from the lower limit of TIVd11, however this QTL was only found to be significant in an additive model with the other two QTLs, but not when considered alone. Another QTL for type IV trichomes is on chromosome 9 in *S. galapagense* explaining 8% of variance [24] and is located 61 Mb away from TIVd9. Therefore, it is unlikely that both QTLs overlap. Moreover, in a posterior study with RIL populations this QTL was not found [64]. However, the chromosomal region of TIVd9 does overlap with a QTL for adult survival to white fly [24]. TIVd5 and TIVd6 overlap with previously detected QTLs for trichome density in *S. pennellii* [21,65]. Additionally TIVd5 was also previously found to be associated with acylsugar accumulation levels [21].

Several types of genes have been found to control the development of trichomes [66,67], including MYB transcription factors that are known to regulate positively and negatively trichome development [68,69], and genes related to the production of acylsugars [56] or plant defenses. Several strong gene candidates for TIVd9 have been found in the QTL region. Besides several enzymes related to acylsugar production, two MYB genes (Solyc09g010820 and Solyc09g009450) are located within the range and especially two trichome birefringence-like proteins (Solyc09g010260 and Solyc09g010270). This gene family influences synthesis of cellulose on the secondary wall, affects resistance and has been shown to regulate the density of trichomes [54,55]. The different gene members are likely to provide a wide range of activities and tissue specificities [55]. In the region of TIVd11 another two MYB R2R3 genes have been found (Solyc11g072060 and Solyc11g073120) and an Agamous-like MADS-box protein (Solyc11g069770). Interestingly, *AGAMOUS* has been shown to negatively interfere with the development of trichomes in *Arabidopsis* [70], which is the same effect found in our population.

The QTLs identified in this work, which increase the density of type IV trichomes and, probably, the synthesis of acylsugars, represent a valuable resource for the development of new tomato varieties. One of the main advantages is that these QTLs have been described in a *S. pimpinellifolium* accession, a species that has been widely and successfully used in tomato breeding and in introgression of alleles. Incorporation of insect resistance into commercial tomatoes is a key point in breeding programs to facilitate the cultivation of crops reducing the use of pesticides. Type IV trichomes could provide broad-spectrum resistance to pests with low impact on tomato fruit as trichomes are not present in fruits. The identification of these QTLs could facilitate the rapid development of new varieties through genetic marker-assisted selection and contribute to the identification of key genes in the control of trichome development.

## 5. Conclusions

The results presented in this study increase the knowledge of the genetic factors involved in trichomes density as several strong candidate regions have been detected, so further studies can address the effect of such genes. The two genetic backgrounds used have detected some different QTL regions, showing the complex mechanism involved in the control of trichome development. Additionally, our results contribute to the creation of tomato lines with a higher density of type IV trichomes, and likely higher insect resistance [16]. Several efforts have been made to develop introgressed lines with type IV trichomes enriched in acylsugars [26,59,61]. We found a major QTL that has not been previously reported in *S. pimpinellifolium* that increases the density of trichomes by a factor of 9 if the alleles are in homozygosis. This accession contains probably different alleles of the QTLs detected in the other species. The main advantage of this accession is that this QTL could be easily introgressed due to the close genetic relationship between *S. pimpinellifolium* and cultivated tomato. Besides, this study is the first example of the utility and efficiency of a new genotyping methodology, K-seq, that with a low read number by sample allows the genotype of millions of bases at low cost. As has been shown, the methodology is easy and robust enough to generate high density maps and to perform QTL-seq.

## Figures and Tables

**Table 1:** Summary statistics for the genetic map built using the F2 generation of the cross BGV016047 × LA2278.

	No. SNPs	No. Bin Markers	Length	Avg. Spacing	Max. Spacing
SL2.50ch01	23,537	1248	153.4	0.1	3.7
SL2.50ch02	10,368	662	130.3	0.2	1.8
SL2.50ch03	8300	479	165.2	0.3	8.5
SL2.50ch04	9482	534	123.9	0.2	1.3
SL2.50ch05	15,374	856	121.4	0.1	7.9
SL2.50ch06	8375	552	123.7	0.2	11.2
SL2.50ch07	17,919	770	110.9	0.1	1.9
SL2.50ch08	7555	563	164.7	0.3	13.3
SL2.50ch09	11,342	725	125.9	0.2	3.7
SL2.50ch10	14,855	788	119.9	0.2	5.2
SL2.50ch11	9925	567	101.8	0.2	2.8
SL2.50ch12	10,294	541	159.2	0.3	6.2
Overall	147,326	8285	1600.4	0.2	13.3

**Table 2:** QTL location based on different methods using SLC family and SLL family mapping populations. For each QTL, chromosome, start and end position, location of the most significant value of the region (Max) and LOD, adjusted *p*-value or ΔSNP value for that location are reported.

**QTL Mapping (SLC Family)**					
**Phenotype**	**Chr**	**Start**	**Max**	**End**	**LOD**
Trichome density 6th week	SL2.50ch09	2,055,839	2,542,786	4,250,325	6.24
	SL2.50ch11	54,298,873	56,297,460	56,297,460	5.62
Trichome density 7th week	SL2.50ch09	2,078,180	3,756,370	4,250,325	6.33
	SL2.50ch11	55,093,420	56,297,460	56,297,460	3.69
Trichome density 8th week	SL2.50ch09	1,966,640	2,309,105	4,371,756	5.36
	SL2.50ch11	55,548,327	56,297,460	56,297,460	3.83
**Association (SLC Family)**					
**Phenotype**	**Chromosome**	**Start**	**Max**	**End**	**Adj. pval.**
Trichome density 6th week	SL2.50ch09	2,309,105	2,542,786	2,545,171	0.0090
Trichome density 7th week	SL2.50ch09	2,078,180	3,727,914	4,969,831	7.7·10^−6^
Trichome density 8th week	SL2.50ch06	8008	154,414	615,838	0.0002
	SL2.50ch09	2,069,769	2,492,691	5,693,896	4.6·10^−5^
**QTLseq**					
**Mapping Population**	**Chromosome**	**Start**	**Max**	**End**	**ΔSNP**
SLC family	SL2.50ch09	3,042,382	3,966,023	4,560,795	0.57
SLL family	SL2.50ch05	9,189,958	33,460,771	59,293,229	0.52
	SL2.50ch06	23,628	23,628	943,353	0.46
	SL2.50ch09	2,105,569	2,865,099	4,800,975	0.56

**Table 3:** Results of the negative binomial logistic regression for the QTLs found by QTL mapping and association analysis. Different statistical models using trichome density (TD) after 6,7 or 8 weeks after transplanting and either a single predictor or an additive model are shown. Independent variables used correspond to the number of alleles of the high density parental at the marker with the highest statistical support. For each model, χ^2^ goodness of fit *p*-value of the model (GOF), Nagelkerke *R*^2^*,* explained deviance (*D*^2^) and point estimated of incidence rate ratio (IRR) and 95% confidence interval are reported. *** *p*-value < 0.001.

**QTL Mapping**							
**Phenotype**	**Model**	**GOF**	***R*^2^**	***D*^2^**	**Variable**	**IRR (95% CI)**	**Sig**
TD 6th w	TIVd9	0.361	0.18	0.16	SL2.50ch09_2542786	2.61 (1.81–3.78)	***
	TIVd11	0.348	0.08	0.07	SL2.50ch11_56297460	0.54 (0.37–0.78)	***
	TIVd9 + TIVd11	0.386	0.30	0.26	SL2.50ch09_2542786	2.79 (1.96–4.03)	***
					SL2.50ch11_56297460	0.47 (0.33–0.67)	***
TD 7th w	TIVd9	0.145	0.26	0.21	SL2.50ch09_3756370	3.24 (2.24–4.72)	***
	TIVd11	0.144	0.09	0.08	SL2.50ch11_56297460	0.49 (0.33–0.73)	***
	TIVd9 + TIVd11	0.149	0.37	0.29	SL2.50ch09_3756370	3.24 (2.28–4.63)	***
					SL2.50ch11_56297460	0.48 (0.34–0.69)	***
TD 8th w	TIVd9	0.084	0.21	0.17	SL2.50ch09_2309105	2.88 (1.98–4.17)	***
	TIVd11	0.076	0.07	0.06	SL2.50ch11_56297460	0.56 (0.38–0.82)	***
	TIVd9 + TIVd11	0.091	0.33	0.26	SL2.50ch09_2309105	3.22 (2.24–4.65)	***
					SL2.50ch11_56297460	0.48 (0.33–0.68)	***
**Association**							
**Phenotype**	**Model**	**GOF**	**R^2^**	**D^2^**	**Variable**	**IRR (95% CI)**	**Sig**
TD 6th w	TIVd9	0.361	0.18	0.16	SL2.50ch09_2542786	2.61 (1.81–3.78)	***
TD 7th w	TIVd9	0.147	0.25	0.20	SL2.50ch09_3727914	3.19 (2.21–4.64)	***
TD 8th w	TIVd6	0.090	0.22	0.17	SL2.50ch06_154414	2.47 (1.79–3.41)	***
	TIVd9	0.085	0.22	0.18	SL2.50ch09_2492691	2.94 (2.03–4.26)	***
	TIVd6 + TIVd9	0.085	0.32	0.25	SL2.50ch06_154414	1.86 (1.33–2.60)	***
					SL2.50ch09_2492691	2.16 (1.44–3.22)	***

## Data Availability

Sequencing data is available through the SRA under Bioproject PRJNA649673. The data presented in this study are available in Supplementary File 2 and Supplementary File 3.

## Supplementary Materials

The following are available online at https://www.mdpi.com/2073-4425/12/2/243/s1, Supplementary Table S1. Genes in the QTL candidate region of chromosome 5, 6, 9 and 11. Number of changes with high, moderate and low impact on gene function as well as those with an uncertain effect (modifier) inferred by SNPEff are shown. Annotation of ITAG4.1 when available is also provided, Supplementary Figure S1. Graphical summary of the experimental design for the evaluation of plant age and leaf age. (A) First and second fully expanded leaves from the apex of the plant. (B) Location of second leaflet of a second fully expanded leaf. (C) Excision of the leaflet perpendicularly to the central vein. Trichome density was estimated in four random areas in the abaxial region, Supplementary Figure S2. Density of type IV trichomes in the matting populations along the three weeks of phenotyping for the SLC family (BGV016047 × LA2278) (A) and SLL family (BGV016047 × MoneyMaker) (B), Supplementary Figure S3. Read number by sample, Supplementary Figure S4. Bases sequenced at 5× and 20× coverages by sample, Supplementary Figure S5. Genotyped SNPs by sample, Supplementary Figure S6 Genetic map of the F2 population of the SLC family based on 8,285 bin markers, Supplementary Figure S7. Results of the association analysis of the trichome IV density along the genome (A) 6th week after transplanting, (B) 7th week and (C) 8th weeks. The minus logarithm of corrected *p*-values obtained after fitting to a negative binomial logistic regression model are shown. Horizontal dashed line represents the *p*-value threshold of 0.01, Supplementary File 1. K-seq primer sequences, Supplementary File 2. Data recorded during trichome density measures. Mean: mean of trichome density from 4 measures per leaf, leafA: first measure, leafB: second measure, leafC: third measure, position: the position of the leaf, counting from the ground to the apex, Supplementary File 3. VCF file with F2 K-seq genotypes.
