# Supplementary material for: Discovery of a Major QTL Controlling Trichome IV Density in Tomato Using K-Seq Genotyping
Source: Genes (Basel). 2021 Feb 8;12(2):243. doi: 10.3390/genes12020243 (PMC7915031; doi:10.3390/genes12020243)
Supplement: Supplementary file 1 [file genes-12-00243-s001.zip › supplemental/Supplementary_file_1.docx]

**Supplemental data 3. Primer sequences**

**Primers K-seq**

adaptF-4T TCG TCG GCA GCG TCA GAT GTG TAT AAG AGA CAG NNN TCA TCT TC

adaptF-5T TCG TCG GCA GCG TCA GAT GTG TAT AAG AGA CAG NNN CAA AGA AG

adaptR-5T GTC TCG TGG GCT CGG AGA TGT GTA TAA GAG ACA GNN NTG TTG ATG

**Primers PCR**

IDT-8nt-NXT_i7_1 CAAGCAGAAGACGGCATACGAGATACGATCAGGTCTCGTGGGCTC*G*G

IDT-8nt-NXT_i7_2 CAAGCAGAAGACGGCATACGAGATTCGAGAGTGTCTCGTGGGCTC*G*G

IDT-8nt-NXT_i7_3 CAAGCAGAAGACGGCATACGAGATCTAGCTCAGTCTCGTGGGCTC*G*G

IDT-8nt-NXT_i7_4 CAAGCAGAAGACGGCATACGAGATATCGTCTCGTCTCGTGGGCTC*G*G

IDT-8nt-NXT_i7_5 CAAGCAGAAGACGGCATACGAGATTCGACAAGGTCTCGTGGGCTC*G*G

IDT-8nt-NXT_i7_6 CAAGCAGAAGACGGCATACGAGATCCTTGGAAGTCTCGTGGGCTC*G*G

IDT-8nt-NXT_i7_7 CAAGCAGAAGACGGCATACGAGATATCATGCGGTCTCGTGGGCTC*G*G

IDT-8nt-NXT_i7_8 CAAGCAGAAGACGGCATACGAGATTGTTCCGTGTCTCGTGGGCTC*G*G

IDT-8nt-NXT_i7_9 CAAGCAGAAGACGGCATACGAGATATTAGCCGGTCTCGTGGGCTC*G*G

IDT-8nt-NXT_i7_10 CAAGCAGAAGACGGCATACGAGATCGATCGATGTCTCGTGGGCTC*G*G

IDT-8nt-NXT_i7_11 CAAGCAGAAGACGGCATACGAGATGATCTTGCGTCTCGTGGGCTC*G*G

IDT-8nt-NXT_i7_12 CAAGCAGAAGACGGCATACGAGATAGGATAGCGTCTCGTGGGCTC*G*G

IDT-8nt-NXT_i5_1 AATGATACGGCGACCACCGAGATCTACACATATGCGCTCGTCGGCAGCG*T*C

IDT-8nt-NXT_i5_2 AATGATACGGCGACCACCGAGATCTACACTGGTACAGTCGTCGGCAGCG*T*C

IDT-8nt-NXT_i5_3 AATGATACGGCGACCACCGAGATCTACACAACCGTTCTCGTCGGCAGCG*T*C

IDT-8nt-NXT_i5_4 AATGATACGGCGACCACCGAGATCTACACTAACCGGTTCGTCGGCAGCG*T*C

IDT-8nt-NXT_i5_5 AATGATACGGCGACCACCGAGATCTACACGAACATCGTCGTCGGCAGCG*T*C

IDT-8nt-NXT_i5_6 AATGATACGGCGACCACCGAGATCTACACCCTTGTAGTCGTCGGCAGCG*T*C

IDT-8nt-NXT_i5_7 AATGATACGGCGACCACCGAGATCTACACTCAGGCTTTCGTCGGCAGCG*T*C

IDT-8nt-NXT_i5_8 AATGATACGGCGACCACCGAGATCTACACGTTCTCGTTCGTCGGCAGCG*T*C

nucleotides labelled with * have a phosphotioate bond to prevent primer degradation by the DNA polymerase
