# Supplementary figures and images for: Discovery of a Major QTL Controlling Trichome IV Density in Tomato Using K-Seq Genotyping
Source: Genes (Basel). 2021 Feb 8;12(2):243. doi: 10.3390/genes12020243 (PMC7915031; doi:10.3390/genes12020243)

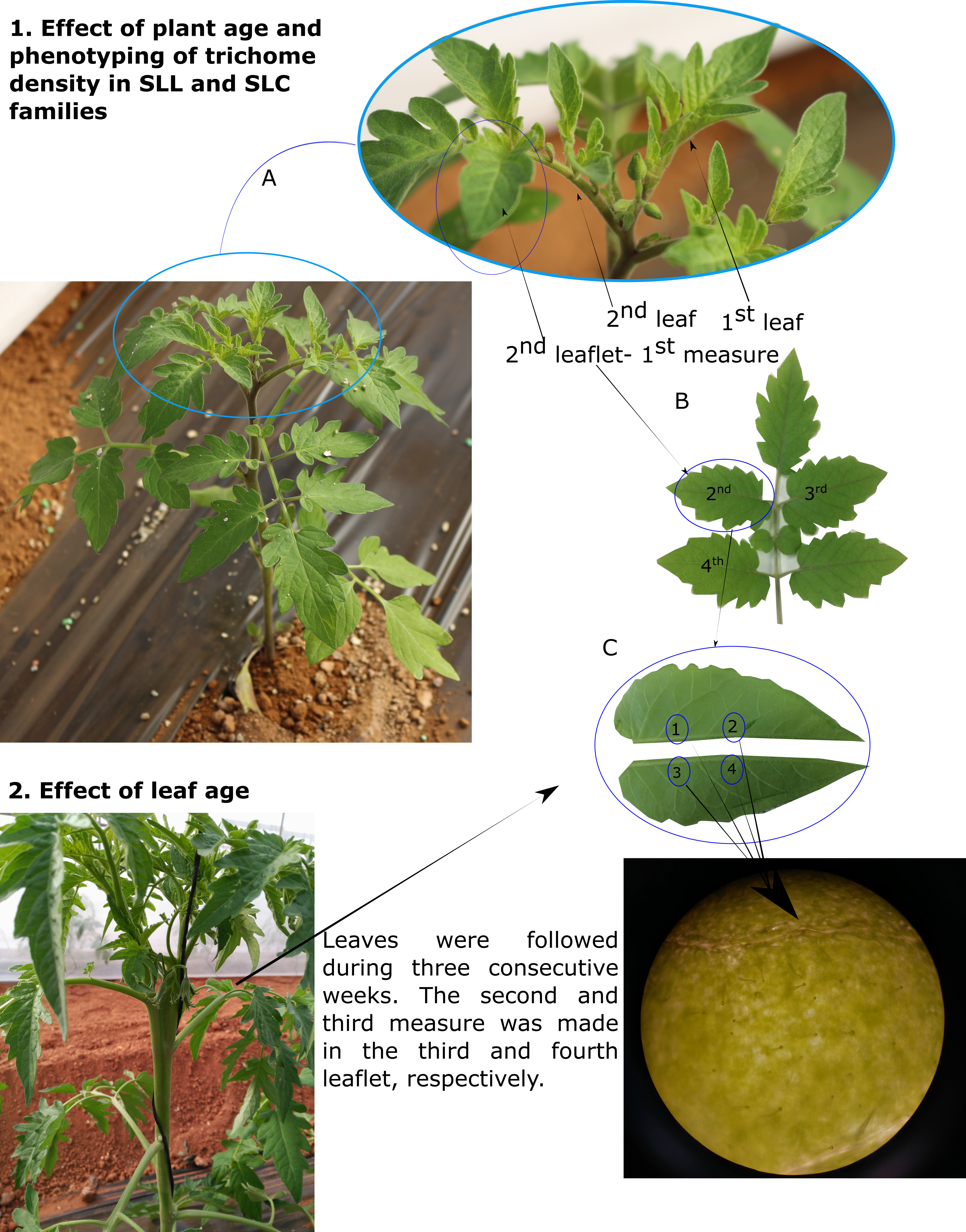

Supplement: Supplementary file 1 [file genes-12-00243-s001.zip › supplemental/Suppl. Fig. 1.png]

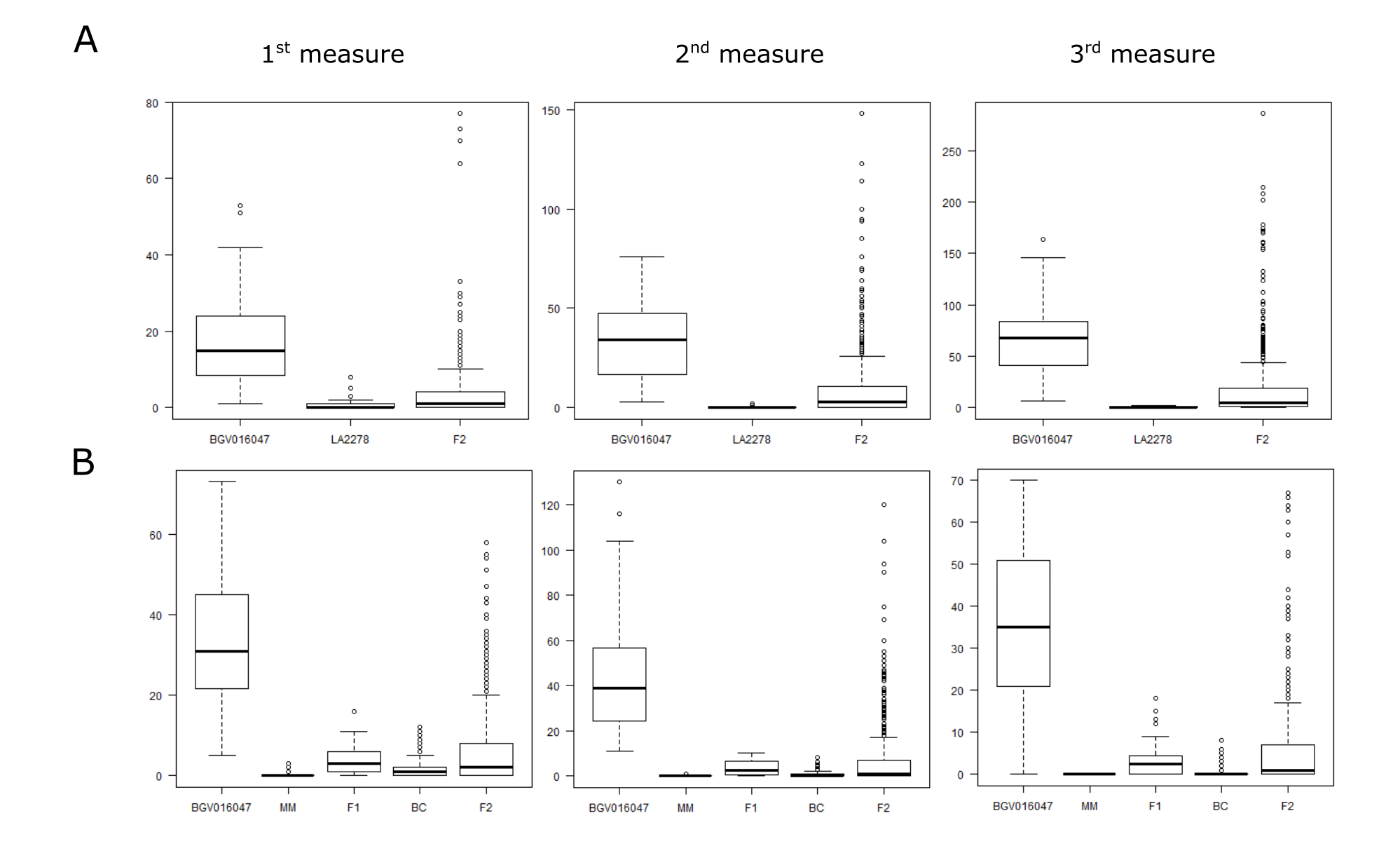

Supplement: Supplementary file 1 [file genes-12-00243-s001.zip › supplemental/Supplementary_Figure2.png]

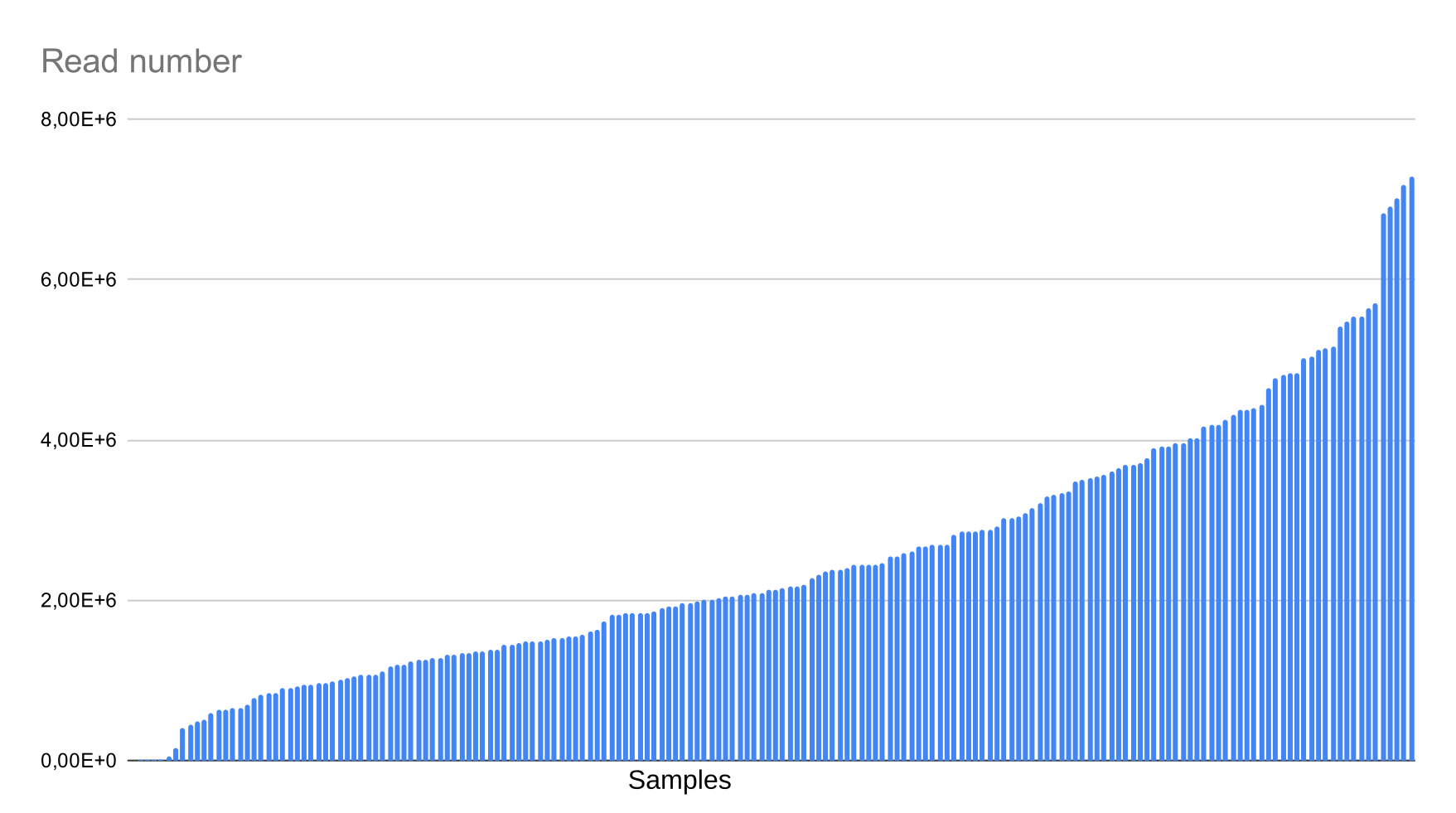

Supplement: Supplementary file 1 [file genes-12-00243-s001.zip › supplemental/Supplementary_figure3.png]

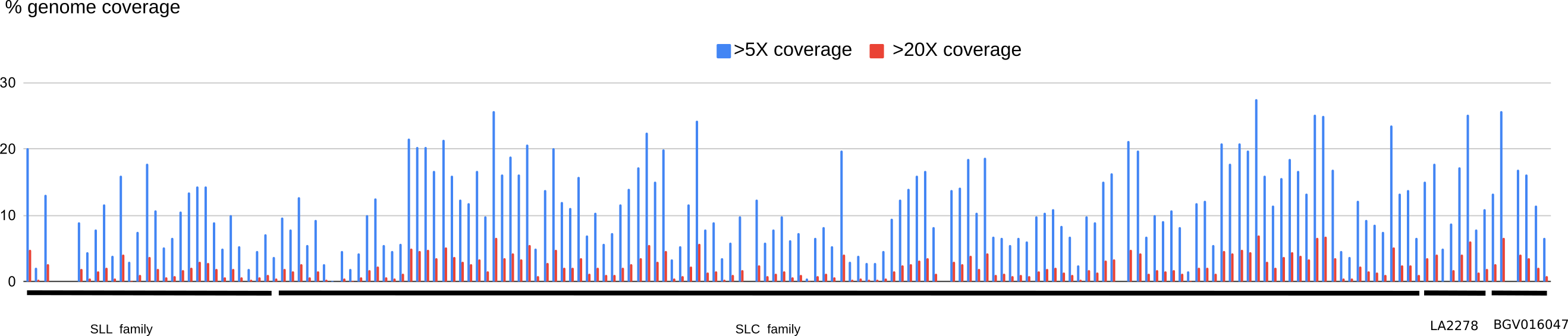

Supplement: Supplementary file 1 [file genes-12-00243-s001.zip › supplemental/Supplementary_figure4.png]

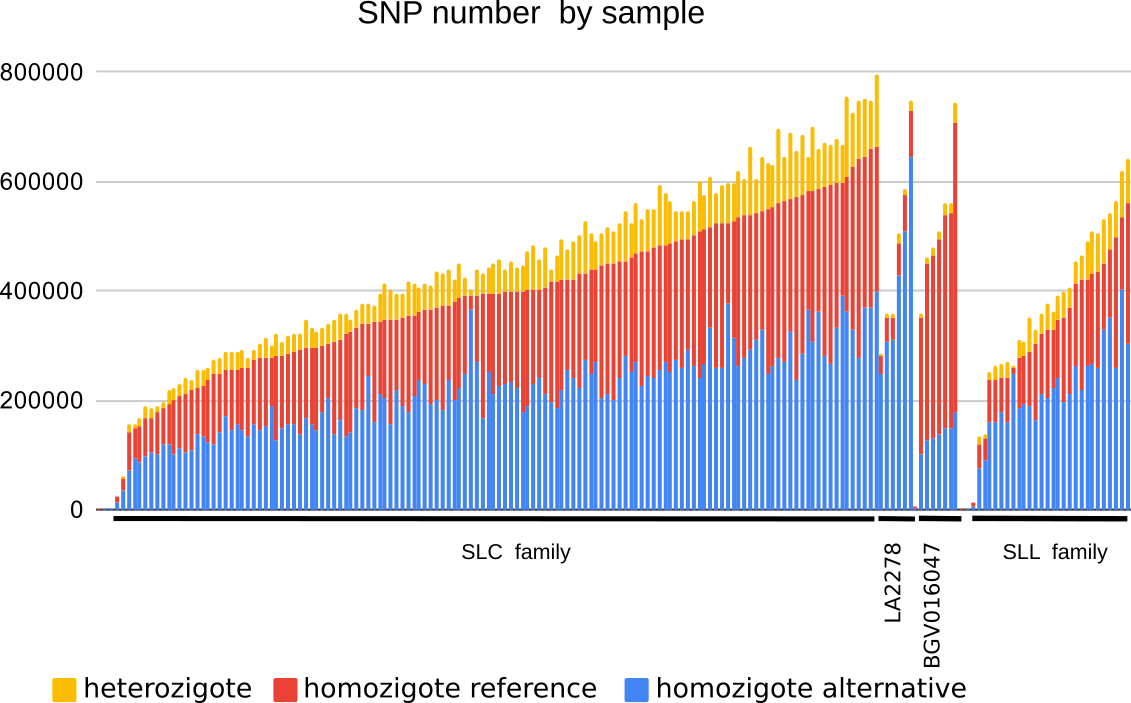

Supplement: Supplementary file 1 [file genes-12-00243-s001.zip › supplemental/Supplementary_figure5.png]

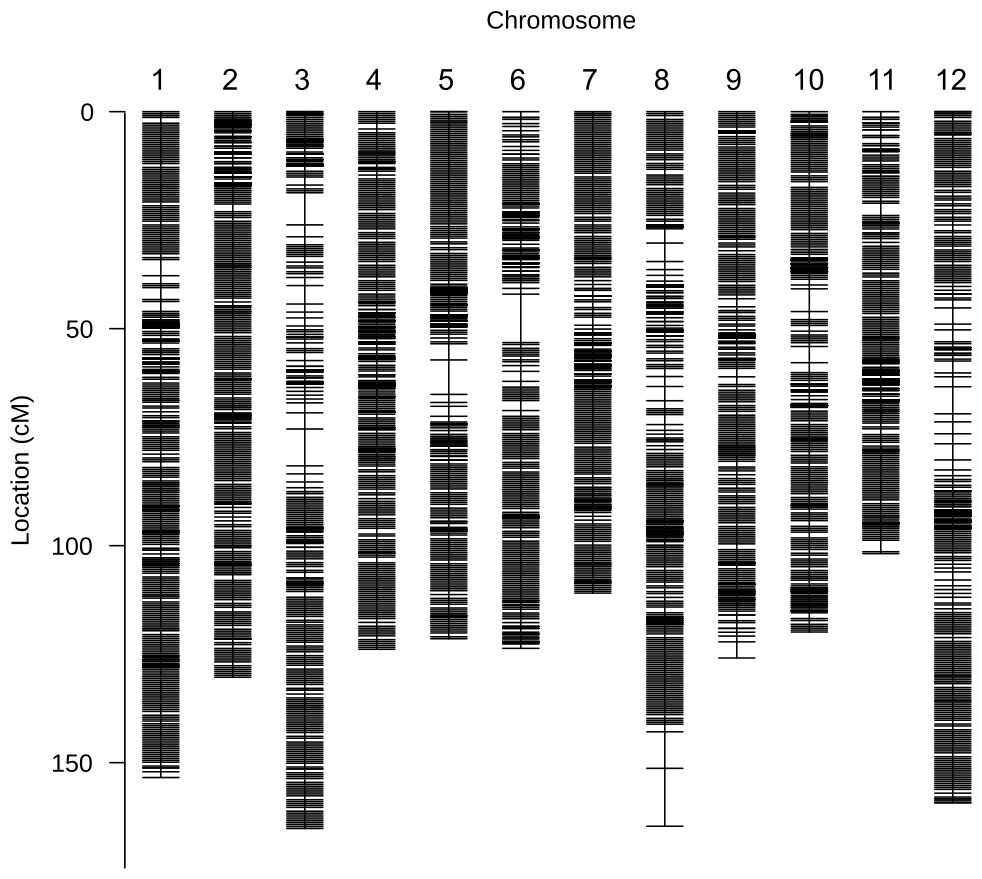

Supplement: Supplementary file 1 [file genes-12-00243-s001.zip › supplemental/Supplementary_Figure6.png]

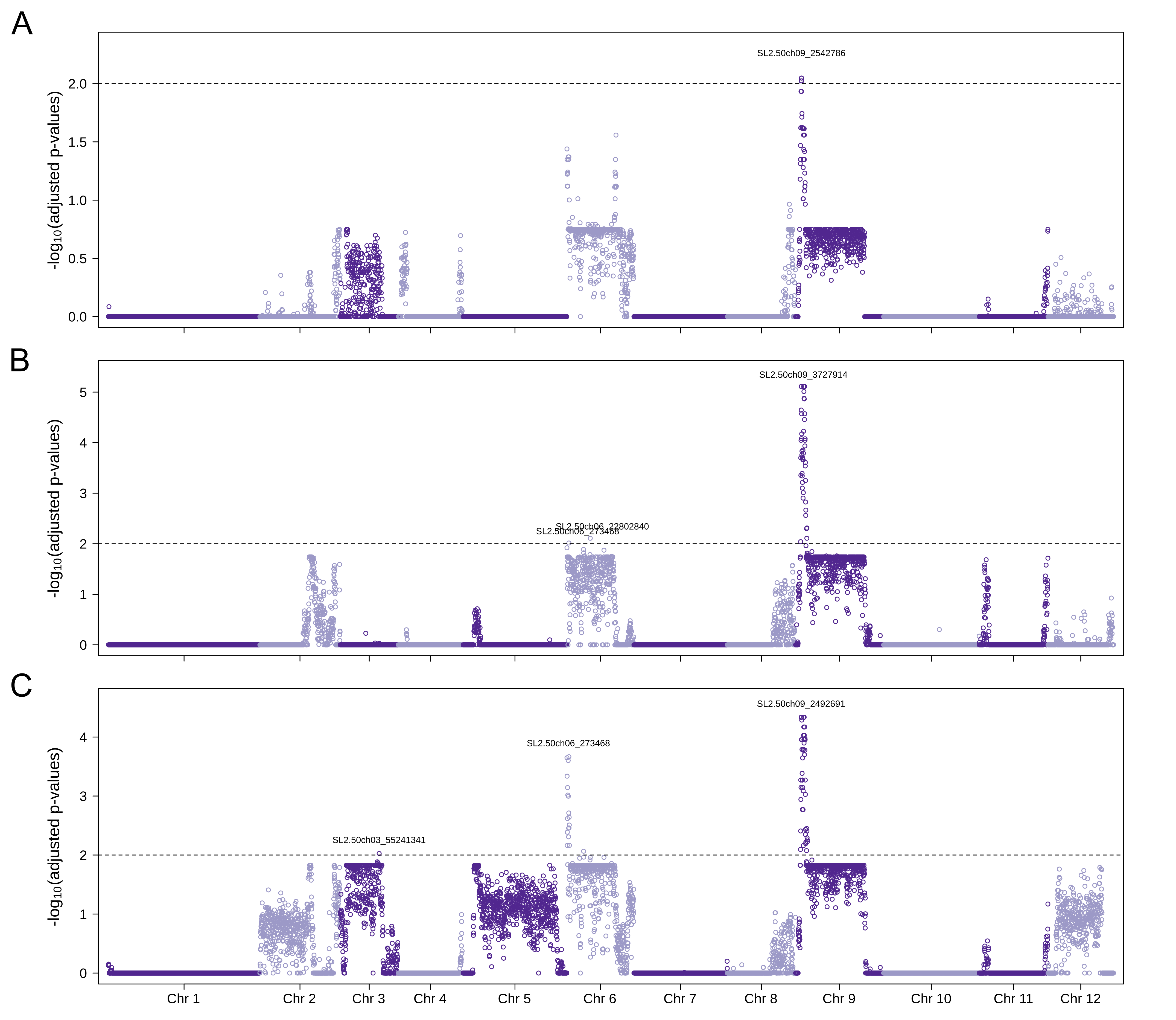

Supplement: Supplementary file 1 [file genes-12-00243-s001.zip › supplemental/Supplementary_Figure7.png]
